# Blood transfusion and coagulopathy in geriatric trauma patients

**DOI:** 10.1186/s13049-017-0374-0

**Published:** 2017-03-29

**Authors:** Brett Mador, Bartolomeu Nascimento, Simon Hollands, Sandro Rizoli

**Affiliations:** 1 Department of Surgery, University of Alberta, 205 – 3017 66 St NW, Edmonton, AB T6K 4B2 Canada; 2 Department of Surgery, Sunnybrook Health Sciences Centre, 2075 Bayview Ave, Room H171, Toronto, ON M4N 3M5 Canada; 3 Institute for Clinical Evaluative Sciences, Sunnybrook Health Sciences Centre, 2075 Bayview Ave, Toronto, ON M4N 3M5 Canada; 4 Department of Surgery, St. Michael’s Hospital, 30 Bond Street, 3-074 Donnelly Wing, Toronto, ON M5B 1W8 Canada

**Keywords:** Geriatric, Trauma, Transfusion, Coagulopathy, Thromboelastography

## Abstract

**Background:**

Trauma resuscitation has undergone a paradigm shift with new emphasis on the early use of blood products and increased proportions of plasma and platelets. However, it is unclear how this strategy is applied or how effective it is in the elderly population. The study aim is to identify differences in transfusion practices and the coagulopathy of trauma in the elderly.

**Methods:**

Data was prospectively collected on all consecutive patients that met trauma activation criteria at a Level I trauma centre. Data fields included patient demographics, co-morbidities, injury and resuscitation data, laboratory values, thromboelastography (TEG) results, and outcome measures. Elderly patients were defined as those 55 and older. Propensity-score matched analysis was completed for patients receiving blood product transfusion. Patients were matched by gender, mechanism, injury severity score (ISS), head injury, and time from injury.

**Results:**

Total of 628 patients were included, of which 142 (23%) were elderly. Elderly patients were more likely to be female (41% vs. 24%), suffer blunt mechanism of trauma (96% vs. 80%), have higher ISS scores (mean 25.4 vs. 21.6) and mortality (19% vs. 8%). Elderly patients were significantly more likely to receive a blood transfusion (42% vs. 30%), specifically for red cells and plasma. Propensity-matched analysis resulted in no difference in red cell transfusion or mortality. Despite the broad similarities between the matched cohorts, trauma coagulopathy as measured by TEG was less commonly observed in the elderly.

**Discussion:**

Our results suggest that elderly trauma patients are more likely to receive blood products when admitted to a trauma centre, though this may be attributed to under-triage. The results also suggest an altered coagulopathic response to traumatic injury which is partially influenced by increased anticoagulant and antiplatelet medication use in the geriatric population.

**Conclusion:**

It is not clear whether the acute coagulopathy of trauma is equivalent in geriatric patients, and further study is therefore warranted.

## Background

Trauma resuscitation has undergone a paradigm shift over the past decade. The benefit of early transfusion of blood products has been realized and changed the approach to the acutely injured, actively bleeding patient [1]. We have also seen the adoption of increased ratios of fresh frozen plasma (FFP) and platelets to packed red blood cells (pRBC), and the development of specific transfusion protocols to corral these efforts [2–4]. Despite limited prospective data there has been wide-spread acceptance of these changes in trauma resuscitation [5]. Furthermore, there is even less data on how these new strategies in transfusion have impacted specific subsets of the trauma population, such as the elderly.

This shift in transfusion practices has largely been driven by an improved understanding of the coagulopathy of trauma, and the discovery that up to a quarter of severely injured trauma patients will arrive to hospital already in a coagulopathic state [6, 7]. This goes beyond the classic triad of hypothermia, hemodilution, and acidosis and involves intrinsic mechanisms such as an accelerated and extensive activation of tissue factor and activated protein C [6, 7]. This leads to a cascade resulting in hypocoagulability and hyperfibrinolysis, which are now recognized as independent predictors of ongoing hemorrhage and mortality [8, 9]. It seems intuitive that this mechanism of trauma coagulopathy would also occur in the elderly populations, though the degree to which this occurs has been scarcely studied.

In order to diagnose acute trauma coagulopathy expeditiously there has been increased utilization of point-of-care coagulopathy testing, or viscoelastic hemostatic assays, such as thromboelastography (TEG) and rotational thromboelastometry (ROTEM). These tests are able to provide rapid determination of coagulation status and help guide transfusion practices [10–12]. However, it is not known whether or not these tests maintain the same accuracy in the elderly population. Scarpelini et al. showed that TEG values in healthy volunteers did not differ between the young and old, though this does not necessarily extrapolate to the traumatized patient [13]. In fact, studies in peri-operative cardiac and orthopedic surgery patients have shown differences in TEG-based coagulation between the young and elderly, even after controlling for pre-existing coagulopathies or anti-coagulant medications [14, 15]. A separate coagulation analysis of healthy volunteers revealed higher concentrations of coagulation factors with age favoring increased fibrin formation and reduced fibrinolysis [16]. Further data also supports enhanced activation of the coagulation system in frail elderly patients [17, 18], though again the impact of these changes in the setting of trauma is lacking and warrants further study.

It has been shown that geriatric patients are frequently under-triaged to trauma centres [19–23]. In fact, in one study elderly patients were half as likely to undergo trauma team activation as younger patients with similar injury severity [19]. This is in spite of evidence showing that the majority of geriatric trauma patients have a chance at achieving good functional outcomes and quality of life following major trauma [24, 25]. This brings to question whether other facets of geriatric trauma care are similarly under-delivered; a critical issue given the well-documented correlation between trauma mortality and age [21].

Our hypotheses are two-fold. First, we hypothesized that geriatric trauma patients are not treated equally when it comes to transfusion of blood products. Specifically, our study aimed to determine how age impacts blood transfusion during trauma resuscitations. It has been shown that geriatric trauma patients tend to be under-triaged, but what is unclear is whether they are under-resuscitated or over-resuscitated, given misleading vital signs secondary to pre-existing medical conditions and medication use [26]. Second, we hypothesized that the acute coagulopathy of trauma varies with age. We sought to specifically identify any differences in trauma coagulopathy in-vivo in the elderly population.

## Methods

A retrospective analysis was performed using prospectively collected data on 790 consecutive patients that met criteria for major trauma activation at a single Level I trauma centre in Toronto, Canada from February to October, 2007. Patients were excluded based on incomplete data, refusal of consent, or declaration of death on arrival. This left 628 patients for further analysis. Data collected included demographics, injury data, vital signs, laboratory results (including TEG), and outcome data including length of stay (LOS), complications, blood product transfusion, and mortality. Primary complications of interest were those associated with blood transfusion, such as thromboembolic disease, infection rates, and acute respiratory distress syndrome (ARDS). Of note, the use of tranexamic acid in trauma patients was not standard practice during the study period and was not administered to any patients.

Elderly was defined as being 55 years of age and older. Studies have shown that mortality tends to increase significantly after 55, 65, and 70 years, and all of these time points (and more) have been used to define elderly in the literature [21, 27, 28]. Ideally, elderly would be defined by the physiologic age at which systemic functions start to decline and response to traumatic injury is impaired. Sadly, defining pre-injury physiologic age for each individual patient is not feasible. Instead, we chose an age cutoff of 55 in compliance with the current American College of Surgeons’ Advanced Trauma Life Support (ATLS) course recommendations for trauma triage [29]. This recommendation is based on population-based data showing a major inflection in trauma mortality at this age [21]. Still, some of the recent literature has moved towards age 65 to define ‘elderly’. For this reason, we performed a pre-planned subgroup analysis using an age cut-off of 65, to confirm that our conclusions would remain relatively consistent when using different definitions of elderly.

Basic descriptive comparisons between groups were performed. We further analyzed the relationship between conventional laboratory tests and TEG values to blood product transfusion. Patients that received at least one unit of blood product transfusion were matched on gender and a propensity score (within 0.2 standard deviations) to one control subject. Patients that did not undergo transfusion were excluded from the matched analysis as the primary population of interest consisted of patients suffering from traumatic hemorrhage and requiring blood transfusion. The propensity score was calculated using the following variables: mechanism of injury, time to hospital, injury severity score (ISS), presence of head injury (defined as head abbreviated injury score or AIS >0), and operative management required. These covariates were chosen because they are directly correlated with blood transfusion [30], with the exception of head injury which was included as it has known effects on important outcomes such as coagulopathy, LOS and mortality [31]. Subjects could serve as a control more than once.

Secondary analysis was performed to determine whether abnormal TEG results could predict the need for blood and blood product transfusion. A growing number of studies is investigating the role of point-of-care coagulopathy testing such as TEG in efforts to optimize transfusion of trauma patients [32]. We investigated whether blinded practitioners would follow the clinical practices as proposed by the 2015 expert consensus conference; for example, whether practicing clinicians without access to TEG results would transfuse FFP to patients in whom TEG would mandate such transfusion. The aim was to explore whether practice patterns align with consensus conference guidelines on blood transfusion in early trauma [12].

Conventional thromboelastography or TEG was utilized during the study but the results were not made available to any of the practitioners involved in the care of the patient, thus no clinical decision was made based on the TEG results. TEG measures the viscoelastic changes as blood clots by spinning a sample of blood through the TEG 5000 Hemostasis Analyzer (Haemoscope Corporation, Niles, IL). Kaolin was added to the citrated blood samples, which were held for 40 min prior to analysis. Process and quality control measures in regards to TEG during the study are as previously described [33]. Normal TEG values were as described by the manufacturer (R [reaction time] 3-8 min; K [kinetic time] 1-3 min; alpha angle [rate of clot formation] 55-78°; MA [maximum amplitude] 51-69mm; LY30 [clot lysis at 30 min] 0-8%). All clinical decisions, including post-admission blood work were done at the discretion of the treating physicians, who were blinded to the TEG results.

Student *t*-test was used to compare continuous data, while Chi-square test was used to compare categorical data between groups. Standardized differences were used initially to compare the balance of baseline characteristics between the two propensity-score matched groups, where a standardized difference less than 0.1 indicates good balance [34]. SAS version 9.3 (SAS Institute, Cary, NC) was used for all analyses and a two-tailed type 1 error rate of 0.05 was used to indicate statistical significance.

## Results

A total of 628 patients met trauma activation criteria and study inclusion criteria. Of these, 142 (22.6%) were classified as elderly while 486 (77.3%) aged <55 years old were controls. General comparison between the two groups is shown in Table 1. Elderly patients were more likely to be female (41% vs. 24%; *p* = 0.0001), suffer blunt mechanism of trauma (96% vs. 81%; *p* = 0.0005) and have higher ISS scores (mean 25.4 vs. 21.6; *p* = 0.003). Elderly patients had a higher incidence of ARDS and pulmonary embolism, but not of other major trauma-related complications. Elderly patients showed a trend towards increased LOS (17.3 vs. 14.1 days; *p* = 0.077) and nosocomial infection rates (30% vs. 24%; *p* = 0.15), though these did not reach statistical significance. Overall, mortality was significantly higher among elderly patients compared to controls (19% vs. 8%; *p* = 0.0001).

**Table 1:** Full cohort demographics and hospital data

Variable	Control (*n* = 486)	Elderly (*n* = 142)	*P* value
Age (mean)	31.54	68.68	<.0001
Male Sex	368 (76%)	84 (59%)	0.0001
Penetrating Mechanism	94 (19%)	6 (4%)	0.001
ISS (mean)	21.64	25.42	0.003
LOS (mean) (days)	13.09	17.30	0.08
ICU Admissions	178 (37%)	51 (36%)	0.94
Mechanic Ventilated (days)	175 (36%)	59 (42%)	0.23
ICU LOS (mean) (days)	6.70	6.17	0.73
Procedure or Operation	258 (53%)	66 (46%)	0.17
Discharged to Rehab	205 (42%)	81 (57%)	0.002
Mortality	37 (8%)	27 (19%)	0.0001
Nosocomial Infection	115 (24%)	42 (30%)	0.15
Stroke	10 (2%)	4 (3%)	0.59
ARDS	8 (2%)	10 (7%)	0.002
Acute Renal Failure	2 (0.4%)	2 (1%)	0.22
Pulmonary Embolism	5 (1%)	5 (4%)	0.05
Myocardial Infarction	0 (0%)	1 (0.7%)	0.98

*ISS* injury severity score, *LOS* length of stay, *ICU* intensive care unit, *ARDS* acute respiratory distress syndrome

Comparison of transfusion rates is shown in Table 2. Significantly more elderly patients received blood transfusions (43% vs. 30%; *p* = 0.012) overall. Elderly patients received significantly more pRBC and FFP. The higher number of elderly patients receiving platelet or cryoprecipitate transfusions did not reach statistical significance compared to controls.

**Table 2:** Full cohort transfusion data

Variable	Control (*n* = 486)	Elderly (*n* = 142)	*P* value
Transfused	147 (30%)	59 (42%)	0.01
Transfused 1st 24 h	97 (20%)	40 (28%)	0.04
Transfused pRBC	146 (30%)	56 (39%)	0.04
Transfused FFP	41 (8%)	21 (15%)	0.03
Transfused Platelets	36 (7%)	15 (11%)	0.23
Transfused Cryoprecipitate	17 (4%)	6 (4%)	0.69

*pRBC* packed red blood cells, *FFP* fresh frozen plasma

Significantly less elderly patients were tachycardic (heart rate >100 beats per minute) on arrival but more of them had significant anemia with hemoglobin levels below 100g/L. More elderly patients were coagulopathic on hospital admission according to elevated international normalized ratio (INR), 11% compared to 3% of controls had INR >1.5 (*p* = 0.0008). However, 23% of the elderly patients with INR >1.5 were taking Coumadin, which could account for the INR findings. Other markers of coagulopathy such as platelet count, fibrinogen serum levels (18.3% vs. 16.7%; *p* = 0.69) and most TEG measurements (27% vs. 33%; *p* = 0.18) were not statistically different when elderly patients were compared to controls, as shown in Table 3. However, elderly were more likely to have abnormalities in MA than controls (19% vs. 11%, *p* = 0.02). When we analyzed the blood transfusion rates in patients with abnormal coagulation tests (TEG or fibrinogen levels), no statistical differences could be detected between young and elderly; results are shown in Table 4.

**Table 3:** Full cohort vital signs, conventional laboratory and TEG values on admission

Variable	Control (*n* = 486)	Elderly (*n* = 142)	*P* value
Heart Rate >100 bpm	192 (40%)	38 (27%)	0.01
Systolic BP <100 mmHg	18 (4%)	9 (6%)	0.18
Temperature <35C	28 (6%)	7 (5%)	0.81
Hemoglobin <100g/L	24 (5%)	19 (13%)	0.0009
Platelet count <100x10^9^/L	12 (3%)	3 (2%)	0.80
Fibrinogen <2.0 g/L	81 (17%)	26 (18%)	0.69
INR > 1.5	15 (3%)	15 (11%)	0.0008
pH <7.2	27 (6%)	9 (6%)	0.97
Base Deficit >6	98 (20%)	28 (20%)	0.24
2+ Abnormal TEG values	60 (12%)	23 (16%)	0.23
Any Abnormal TEG value	133 (27%)	47 (33%)	0.18
Abnormal R	57 (12%)	11 (8%)	0.16
Abnormal K	51 (10%)	21 (15%)	0.18
Abnormal alpha	74 (15%)	18 (13%)	0.39
Abnormal MA	55 (11%)	27 (19%)	0.02
Abnormal LY30	10 (2%)	3 (2%)	0.98

*INR* international normalized ratio, *TEG* thromboelastography, *R* reaction time, *K* kinetic time, *MA* maximum amplitude, *LY30* clot lysis after 30 minutes

**Table 4:** Patients with abnormal TEG or fibrinogen values on admission

Variable	Control (*n* = 486)	Elderly (*n* = 142)	*P*-value
Any Abnormal TEG value	60 (12%)	23 (16%)	0.26
Any transfusion	24 (40%)	11 (48%)	0.52
Transfused pRBC	24 (40%)	11 (48%)	0.52
Transfused FFP	13 (22%)	4 (17%)	0.67
Transfused Platelets	13 (22%)	4 (17%)	0.67
Transfused Cryoprecipitate	7 (12%)	1 (4%)	0.33
Abnormal LY30	10 (2%)	3 (2%)	0.98
Any transfusion	8 (80%)	2 (67%)	0.63
Transfused Cryoprecipitate	6 (60%)	0 (0%)	
Mortality	6 (60%)	3 (100%)	
Abnormal Fibrinogen	81 (17%)	26 (18%)	0.69
Any transfusion	53 (65%)	20 (77%)	0.28
Transfused Cryoprecipitate	13 (16%)	4 (15%)	0.94

*TEG* thromboelastography, *pRBC* packed red blood cells, *FFP* fresh frozen plasma, *LY30* clot lysis after 30 minutes

The next investigation was whether practicing clinicians without access to point-of-care TEG results would transfuse blood products to patients in whom the TEG test would recommend transfusion according to the recommendations of the 2015 expert consensus conference [12]. Any abnormal TEG was associated with the transfusion of pRBC in 42.2% of patients, though was associated with the need for FFP, platelets, or cryoprecipitate in only 20.5%, 20.5%, and 9.6% respectively. In this study we observed a discrepancy between the practitioner decision to transfuse blood products and the recommendations of the 2015 consensus guidelines, in which cryoprecipitate should be transfused for abnormalities in clot strength, such as TEG MA [12]. Similarly, abnormal serum fibrinogen levels or TEG LY30 on admission were predictive of any blood transfusion (68.2%, 76.9%), but not cryoprecipitate specifically (15.9%, 46.2%), which was the standard treatment for hypofibrinogenemia at our institution during the study period. Patients with abnormal LY30 on admission had mortality rates of 60% in the younger cohort and 100% in the elderly, which is consistent with current data regarding this high mortality group [9].

Propensity-matched analysis results are shown in Tables 5 and 6, where 61 elderly were matched to 61 control subjects. After adjustment, a number of the previously observed differences between controls and elderly were resolved, including need for pRBC transfusion, INR >1.5, tachycardia, and mortality. Mortality results were directly compared in the matched cohort, and causes of death were similar between groups with equal proportions of deaths being attributable to bleeding complications.

**Table 5:** Propensity-score matched patient outcomes and transfusion data

Variable	Control (*n* = 61)	Elderly (*n* = 61)	*P*-value
ICU Admissions	41 (67%)	37 (61%)	0.52
Mechanically Ventilated	44 (72%)	40 (66%)	0.43
Discharged to Rehab	27 (44%)	40 (66%)	0.02
Mortality	22 (37%)	17 (28%)	0.33
Nosocomial Infection	19 (31%)	30 (49%)	0.04
Stroke	11 (18%)	3 (5%)	0.03
ARDS	1 (2%)	8 (13%)	0.04
Acute Renal Failure	0 (0%)	2 (3%)	0.99
Pulmonary Embolism	0 (0%)	4 (7%)	0.98
Transfused	55 (90%)	57 (93%)	0.51
Transfused 1st 24 h	31 (51%)	38 (62%)	0.20
Transfused pRBC	55 (90%)	54 (89%)	0.77
Transfused FFP	8 (13%)	20 (33%)	0.01
Transfused Platelets	6 (10%)	14 (23%)	0.06
Transfused Cryoprecipitate	2 (3%)	5 (8%)	0.26

*ICU* intensive care unit, *ARDS* acute respiratory distress syndrome, *pRBC* packed red blood cells, *FFP* fresh frozen plasma

**Table 6:** Propensity-score matched vital signs, conventional laboratory and TEG values on admission

Variable	Control (*n* = 61)	Elderly (*n* = 61)	*P*-value
Heart Rate >100 bpm	22 (36%)	16 (26%)	0.16
Systolic BP <100 mmHg	7 (11%)	8 (13%)	0.89
Temperature <35C	11 (18%)	5 (8%)	0.11
Hemoglobin <100g/L	9 (15%)	17 (28%)	0.08
Platelet count <100x10^9^/L	5 (8%)	2 (3%)	0.26
Fibrinogen <2.0g/L	27 (44%)	20 (33%)	0.05
INR > 1.5	11 (18%)	11 (18%)	0.86
pH <7.2	11 (18%)	5 (8%)	0.09
Base Deficit >6	28 (46%)	17 (28%)	0.01
2+ Abnormal TEG values	12 (20%)	12 (20%)	1.00
Any Abnormal TEG value	29 (48%)	22 (36%)	0.20
Abnormal R	14 (23%)	5 (8%)	0.03
Abnormal K	12 (20%)	11 (18%)	0.66
Abnormal alpha	20 (33%)	7 (11%)	0.002
Abnormal MA	10 (16%)	14 (23%)	0.51
Abnormal LY30	1 (2%)	2 (3%)	0.61

*INR* international normalized ratio, *TEG* thromboelastography, *R* reaction time, *K* kinetic time, *MA* maximum amplitude, *LY30* clot lysis after 30 minutes

There was an increased rate of FFP transfusion (33 vs. 13%; *p* = 0.012), though 6 of the 20 elderly patients that received FFP were on Coumadin. The younger cohort were more likely to have a base deficit <6 and a fibrinogen level <2.0. The elderly were more likely to be discharged to a rehabilitation facility, though mortality differences were not statistically significant. Abnormalities in TEG data for reaction time and alpha angle were more common in the younger cohort.

Subgroup analysis using an age cut-off of 65 years to define elderly was also performed, specifically to determine whether our chosen age cut-off of 55 was appropriate (data not shown). Cohort sizes were not felt to be large enough to allow for meaningful comparisons between more selective age subgroups. The new elderly cohort consisted of 88 patients. Only a few changes were found when compared to the 55 year cut-off. Increased rates of platelet transfusion and LOS in the elderly reached statistical significance in this analysis. For the propensity-matched analysis of this subgroup, the only major difference from the previous analysis was an increased mortality rate in the elderly population (36 vs. 16%, *p* < 0.05).

## Discussion

The main findings of the present study are that, in un-adjusted analysis of all trauma activations at a single institution, elderly patients tend to be more severely injured with higher rates of blood transfusion and mortality. Propensity-matched analysis was performed to specifically analyze the role of TEG in this population and resolved many of the differences between cohorts, while also suggesting a decreased rate of acute trauma coagulopathy in the elderly.

Our results endorse earlier findings, particularly that elderly patients have worse clinical outcomes after trauma. This was true in regards to complications, rehabilitation requirements and mortality. However, this needs to be taken in light of the fact that elderly patients in our cohort were also more severely injured, with a mean injury severity score of 25.4 vs. 21.6 (*p* = 0.003). While higher ISS could account for the higher mortality, it may not be the sole explanation. Another explanation for the higher observed mortality is the increased utilization of blood products in the elderly cohort. Higher transfusion volumes have been associated with mortality in trauma patients even when controlling for injury burden [35], and this effect may be even more pronounced in older patients.

Furthermore, the higher ISS measurements in the elderly supports the findings from American trauma databases [19–23] that older patients are frequently under-triaged to tertiary trauma centres; a major problem considering that geriatric patients tend to do worse when admitted to non-trauma centres. In Florida they were able to show statistically significant improvements in mortality when admitted to a designated trauma centre [36]. Our data supports advocacy for improved triage of elderly patients, even with relatively normal physiology and seemingly benign mechanisms of injury.

In terms of transfusion practices, elderly patients seem to be transfused more readily than their younger counterparts. Statistically significant differences in ISS, INR, and anemia rates may account for this. Elderly patients are also more likely to have more co-morbidities and use of anticoagulants and/or antiplatelet agents (20% vs. 1% in this study). It has also been well established that elderly patients have decreased physiologic reserve and present with less reliable vital signs [26, 37]. Despite normal admission vitals, elderly patients are more prone to decompensation over time; clinicians may therefore more aggressively resuscitate these patients, particularly in the period before internal bleeding has been reliably excluded. In addition, elderly patients may externally appear unwell due to non-trauma related baseline changes such as frailty, age-related skin changes, and co-morbidities. At times these factors lead to inappropriate transfusion decisions in the elderly.

Outside the acutely injured population, altered coagulopathy in the elderly has been previously studied, although conclusions have been conflicting. Roeloffzen et al. showed that baseline TEG values may actually vary with age, showing that elderly patients were more hypercoagulable [38]. However, this baseline hypercoagulability was not confirmed by a larger study using normal subjects performed at our own institution [13]. Age-based differences in TEG has also been looked at in peri-operative patients, though again the data was conflicting, with one study favoring a hypocoagulable state in the elderly versus hypercoagulability in another [14, 15].

To our knowledge this is the first study to look specifically at alterations in TEG following acute trauma in the elderly. After controlling for gender, mechanism, time from injury, head injury, injury severity, and need for operative intervention, we found a significant difference favoring the early development of trauma coagulopathy in younger patients. Despite similar statistics in this propensity-matched cohort, including admission vitals, transfusion, and mortality, the young still showed more frequent abnormalities in reaction time and alpha angle on TEG analysis, and lower fibrinogen levels. This trend persisted using an age cutoff of 65, though with a shift in mortality favoring the young. So, while elderly patients do seem to develop a similar coagulopathy after trauma, this appears less pronounced possibly due to a baseline increase in clotting factor concentration [16]. Unfortunately, our data includes only a minority of patients that required a massive transfusion and is therefore underpowered to comment on relationships between blood transfusion and outcome in this high risk group. However, based on our results further study seems warranted.

If our results are confirmed it will have significant impact on resuscitation practices in a growing trauma sub-population. We observed an increase in trauma-induced coagulopathy in the young, compared to drug-induced coagulopathy in the elderly. Unfortunately, drug-induced coagulopathy is not always diagnosed using viscoelastic testing such at TEG [12]. This would suggest that our approach in the elderly should be more focused on obtaining accurate medication histories and the use of prothrombin complex concentrate, vitamin K, and other reversal agents. Viscoelastic hemostatic assays have proven efficacy in reducing FFP and platelet transfusions in hepatic transplantation, cardiac surgery [39, 40], and most recently trauma populations [35]. Given our results, however, the utility and potential pitfalls of these point-of-care coagulation tests in the geriatric trauma population are uncertain. Specifically, the interaction between anticoagulant medications and the acute coagulopathy of trauma has not been previously addressed and has the potential to alter management in the elderly trauma population. Similarly, what affect do major comorbidities such as heart and renal failure have on the acute coagulation of trauma and viscoelastic testing? A prospective, multicenter study utilizing viscoelastic and conventional coagulation testing would be ideal to further clarify coagulopathy and consequent management of the elderly trauma patient.

This study has a number of limitations. First, there is no clear definition for “elderly”. We chose 55 as a cut-off based on the understanding that physiologic changes occur around this age that impair many patient’s tolerance to major trauma. Furthermore, subgroup analysis of those aged 65 and over did reveal similar trends, albeit with a smaller sample size. Second, our data is made up of a heterogeneous group of patients, as with any trauma database. Propensity-matching helped to limit the differences between the groups, but this remains a difficult factor to minimize. For example, differences in bone density and other underlying co-morbidities are not always clearly defined in the data. Third, the decision-making process in regards to transfusion is not documented. We know that the clinicians did not have access to the TEG data at the time of treatment, but it remains unclear whether each individual unit was ordered based on vital signs, medical history, laboratory data, or other clinical information. Furthermore, the transfusion practices in place at the time of the study may not be characteristic of those used today. Changes in institutional transfusion protocols implemented over this time frame include the now routine use of tranexamic acid in bleeding trauma patients, massive transfusion protocols, and quality improvement initiatives. Finally, this study was performed at a single centre and used only conventional TEG as opposed to rapid TEG or ROTEM, which limits its external validity.

Despite its limitations, this remains the first study to our knowledge in the literature to look specifically at modern day transfusion practices and TEG-based coagulopathy in the geriatric trauma population. Our results show that elderly patients at our trauma centre are more likely to be transfused pRBC and FFP, though this may be due to undertriage and increased anemia rates. Also, elderly patients seem to be less susceptible to alterations in TEG and therefore trauma-induced coagulopathy. While we have shown, in another way, that geriatric patients are different, there are still many gaps to fill in to optimize resuscitation in this high-risk patient group.

## Conclusions

Our results suggest that elderly trauma patients are more likely to receive blood products when admitted to a trauma centre, though this may be attributed to under-triage. It is not clear whether the pathophysiology of coagulopathy in trauma is equivalent in geriatric patients, or consequently its management, and further study is therefore warranted.

## Abbreviations

AIS: Abbreviated injury score
ARDS: Acute respiratory distress syndrome
ATLS: Advanced trauma life support
FFP: Fresh frozen plasma
ICU: Intensive care unit
INR: International normalized ratio
ISS: Injury severity score
k: Kinetic time
LOS: Length of stay
LY30: Clot lysis after 30 minutes
MA: Maximum amplitude
pRBC: packed red blood cells
R: Reaction time
ROTEM: Rotational thromboelastometry
TEG: Thromboelastography
